# Evaluating the impact of neurosurgical rotation experience in Africa on the interest and perception of medical students towards a career in neurosurgery: a protocol for a continental, cross-sectional study

**DOI:** 10.1093/jsprm/snac006

**Published:** 2022-04-02

**Authors:** Setthasorn Zhi Yang Ooi, Olaoluwa Ezekiel Dada, George William Bukenya, Yves Jordan Kenfack, Chi Le, Efosa Ohonba, Emmanuel Adeyemo, Kapil Narain, Ahmed Khaled Awad, Umaru Barrie, Dawin Sichimba, Oloruntoba Ogunfolaji, Lilian Mwende Kitonga, Adaeze Juanita Oriaku, Michael Abayomi Bamimore, Douglas Emeka Okor, Ola Rominiyi

**Affiliations:** School of Medicine, Cardiff University, Cardiff, UK; College of Medicine, University of Ibadan, Ibadan, Nigeria; School of Medicine, Case Western Reserve University, Cleveland OH, USA; School of Medicine, University of Texas Southwestern Medical Center, TX, USA; School of Medicine, Vanderbilt University, Tennessee, USA; Department of Health, North West Province, Mahikeng, South Africa; School of Medicine, University of Texas Southwestern Medical Center, TX, USA; Nelson R. Mandela School of Medicine, University of KwaZulu-Natal, Berea, South Africa; Faculty of Medicine, Ain Shams University, Cairo, Egypt; School of Medicine, University of Texas Southwestern Medical Center, TX, USA; Michael Chilufya Sata School of Medicine, Copperbelt University, Kitwe, Zambia; College of Medicine, University of Ibadan, Ibadan, Nigeria; College of Health Sciences, School of Medicine, University of Nairobi, Nairobi, Kenya; Glan Clwyd Hospital, Betsi Cadwaladr University Health Board, Wales, UK; School of Medicine, Philadelphia College of Osteopathic Medicine, Philadelphia, PA, USA; Department of Neurosurgery, Garki Hospital, Abuja, Nigeria; Department of Neurosurgery, Sheffield Teaching Hospitals NHS Foundation Trust, Sheffield, UK; Department of Neuroscience, University of Sheffield, Sheffield, UK

## Abstract

**Introduction:**

Africa has the second highest neurosurgical workforce deficit globally. Despite the many recent advancements in increasing neurosurgical access in Africa, published reports have shown that the vast majority of undergraduate students have little or no exposure to neurosurgery. The lack of exposure may pose a challenge in reducing the neurosurgical workforce deficit, which is one of the long-term strategies of tackling the unmet burden of disease. Students may also miss the opportunity to appreciate the specialty and its demands as well as nurture their interest in the field. This study aims to assess the impact of a neurosurgical rotation during medical school in shaping the perception and interest of students towards a career in neurosurgery.

**Methods:**

The cross-sectional study will be conducted through the dissemination of a self-administered e-survey hosted on Google Forms from 21st February 2021 to 20th March 2021. The survey will contain five-point Likert scale, multiple-choice and free-text questions. The structured questionnaire will have four sections with 27 items: (i) socio-demographic background, (ii) neurosurgical experience, (iii) perception towards a neurosurgical career and (iv) interest in a neurosurgical career. All consenting medical students in African medical schools who are in their clinical years (defined as fourth to sixth years or higher years of study) will be eligible. Odds ratios and their 95% confidence intervals, Wilcoxon rank-sum test, Welch *t*-test and adjusted logistic regression models will be used to test for associations between independent and dependent variables. Statistical significance will be accepted at *P* < 0.05.

## INTRODUCTION

According to a 2016 analysis, neurological disorders are the leading cause of disability-adjusted life years (DALYs) lost and the second leading cause of death globally [1]. The global burden of the disease is largely concentrated in low- and middle-income countries (LMICs; [2, 3]) and is particularly apparent in the neurosurgical landscape seen in Africa.

Africa has the second highest neurosurgical workforce deficit reported globally [2], despite recent advancements in increasing the number of training centers and increased collaboration with international neurosurgical foundations and neurosurgery departments from developed countries [4]. The deficit is attributed to the small ratio of neurosurgeons per capita, and the difficulty in accessing neurosurgical care within the optimal time frame [5]. This deficit has impacted surgical capacity negatively, with an estimated 1 877 568 surgical case deficit annually [2]. Resolving this unmet need is critical to ensuring timely access to high-quality care, reducing complication rates and ultimately improving the quality of life of patients.

Recently published literature reported that the vast majority of undergraduate students in Africa have little or no exposure to neurosurgery [6]. This is particularly worrying as the scarcity of avenues for students to gain exposure to the specialty may limit opportunities for them to nurture interest in the field and to gain insight into what it entails. Research has shown that exposure to a specialty through clinical rotations positively impacts students’ decision to pursue a career in the specialty [7]. Having time in theatres, access and communication with surgeons, professional relationships and mentor–mentee relationships, amongst others, also play a role in this positive experience [8]. Adequate exposure to neurosurgery is crucial to inspire the next generation of neurosurgeons and to ensure the specialty attracts the best candidates that would make positive changes to the field. Therefore, the lack of exposure may pose a challenge in reducing the neurosurgical workforce deficit, which is one of the long-term strategies of tackling the unmet burden of disease.

To our knowledge, there is a paucity of studies appraising the delivery of neurosurgical education and exposure at undergraduate level in Africa. Its potential impact on the perceptions and interest of medical students towards neurosurgery as a potential career has also yet to be evaluated. We therefore propose a cross-sectional study to address this knowledge gap.

## METHODS AND ANALYSIS

### Study design

This cross-sectional study will use a self-administered e-survey to collect data on neurosurgical exposure and perceptions of and interest in a career in neurosurgery from medical students in Africa. A team of neurosurgeons and medical students will be consulted to assess the questionnaire’s face validity and to ensure the avoidance of any potentially confusing terms. A pilot survey will also be distributed to 15 randomly selected clinical medical students in Africa, who are not involved in the conception or design of the study, to seek feedback, improve clarity and ensure objectivity prior to full administration of the cross-sectional survey.

### Eligibility criteria

#### Inclusion criteria

- All consenting medical students studying in Africa and who are in their clinical years (defined as fourth to sixth year or higher year of study).

#### Exclusion criteria

- Medical students studying in Africa and who are in their preclinical years (defined as first to third years). This group will be excluded because they would have not had formal clinical rotations arranged by the medical school.

### Data collection tools and technique

A 27-item, self-administered structured Google Form (Google, CA, USA) electronic survey in French and English will be used for the study (see online Supplementary material). The questionnaire will include five-point Likert scales, multiple-choice and free-text questions to improve the granularity of the data collected. All initial questions in the survey required a response to minimize any potential missing data at submission. The survey will be disseminated between 21st February 2021 and 20th March 2021. The structured questionnaire will have four sections (A–D). They are:

Section A—Socio-demographic background.

Section B—Neurosurgical exposure of respondents.

Section C—Perception towards a neurosurgical career.

Section D—Interest in a neurosurgical career.

Written consent will be obtained from the respondents before the administration of questionnaires. Questions under the perception towards a neurosurgical career category were adapted from Zuckerman *et al*. [9].

#### Sample size

It is a challenge to establish the minimum sample size required for the survey given that there is no published data on the current number of medical students enrolled in African medical schools.

#### Outcome measures

The primary outcome is the perception and interest of medical students in Africa on pursuing a career in neurosurgery.

Secondary outcomes will include the exposure of medical students to neurosurgery and its correlation with the perception and interest of students. Exposure will be categorized into formal, informal and overseas. Formal neurosurgical exposure is defined as having experienced a neurosurgical clinical rotation regardless of whether it was arranged by the medical school or self. Informal exposure is defined as experience sought outside of a clinical rotation. Overseas neurosurgical exposure is defined as having experienced a neurosurgical clinical rotation or elective outside Africa.

#### Data analysis

Independent variables will include age, sex, country, year of study, urban/rural location of medical school and previous exposure to neurosurgery. Dependent variables will include the perception and interest of the medical students to pursue a career in neurosurgery.

Data will be analyzed using STATA 16.1 (Stata, Version 16.1, StataCorp, USA). Descriptive statistics will be performed for all variables. Depending on normality, the median and interquartile range or mean and 95% confidence interval (CI) were calculated for each domain of perception toward neurosurgery among rotators (defined as students who have undertaken a formal neurosurgical clinical rotation) and non-rotators. Differences in each domain of perception toward neurosurgery were analyzed using the Wilcoxon rank-sum test for non-parametric variables and the Welch *t*-test for parametric variables. Adjusted logistic regression models were developed to estimate the odds of definite interest in neurosurgery careers. Sequential addition of covariates (age, gender and geographical location) and likelihood ratio tests of nested models were conducted to identify the model of best fit. The model examined previous formal neurosurgery rotation and length of formal neurosurgery exposure (more than 4 weeks vs. 4 weeks or less). The 4-week cut-off was common practice for the minimum length of clinical rotations in medical schools worldwide [7]. The final models were adjusted for gender. The interaction between geographical location and formal rotation experience was detected and included. Statistical significance was accepted at *P* < 0.05.

## ETHICS AND DISSEMINATION

The National Health Service (NHS) Health Research Authority decision tool (available at: http://www.hra-decisiontools.org.uk/research/) was applied to establish whether or not specific research ethics approval was required for the study. This tool confirmed that specific research ethics approval was not required. All data will be anonymized, with informed consent taken from all participants. The work will be carried out in accordance with the Declaration of Helsinki, including, but not limited to the anonymity of participants being guaranteed and the informed consent of participants being obtained. We plan to publish this review in a peer-reviewed journal. We may also present this review at local and/or international conferences.

## AUTHORS’ CONTRIBUTIONS

OED and OR contributed to the conception and design of the study. SZYO drafted the manuscript. OR supervised the study process. All authors revised the manuscript critically for important intellectual content and approved for the manuscript to be published. SZYO and OED contributed equally to this work and share first authorship.

## Supplementary Material

Supplementary_-_JSPRM_snac006
