# Supplementary material for: Evaluating the impact of neurosurgical rotation experience in Africa on the interest and perception of medical students towards a career in neurosurgery: a protocol for a continental, cross-sectional study
Source: J Surg Protoc Res Methodol. 2022 Apr 2;2022(2):snac006. doi: 10.1093/jsprm/snac006 (PMC9027747; doi:10.1093/jsprm/snac006)
Supplement: Supplementary_-_JSPRM_snac006 [file supplementary_-_jsprm_snac006.docx]

**Supplementary.** This table shows the list of questions included in the 27-item questionnaire that will be distributed to the medical students.

| **INTEREST AND PERCEPTION OF CLINICAL MEDICAL STUDENT TOWARDS A NEUROSURGICAL CAREER**    This survey is only meant for clinical medical students in African universities.  This survey will help us to ascertain the impact of a neurosurgery rotation and lack of neurosurgery rotation on the perception of clinical students towards a neurosurgical career. Data collected from this survey is non-identifiable and will be used for research purposes.    ** Required* |
| --- |
| **Question 1**  By filling this form, you consent that you understand and agree with the terms stated above. *  ● Yes  ● No  *(Multiple choice)* |
| **Section A**: Sociodemographic background of respondents |
| **Question 2**  Gender *  ● Female  ● Male  ● Non-binary  ● Prefer not to say  *(Multiple choice)* |
| **Question 3**  Age (years) *  ● 15-20  ● 21-25  ● 26-30  ● 31-35  ● 36-40  ● >40  *(Multiple choice)* |
| **Question 4**  Country of study *  ● Algeria  ● Angola  ● Benin Republic  ● Botswana  ● Burkina Faso  ● Burundi  ● Cabo Verde  ● Cameroon  ● Central African Republic (CAR)  ● Chad  ● Comoros  ● Congo  ● "Democratic Republic of the Congo"  ● "Republic of the Côte d'Ivoire"  ● Djibouti  ● Egypt  ● Equatorial Guinea  ● Eritrea  ● Eswatini(Swaziland)  ● Ethiopia  ● Gabon  ● Gambia  ● Ghana  ● Guinea-Bissau  ● Guinea Conakry  ● Kenya  ● Lesotho  ● Liberia  ● Libya  ● Madagascar  ● Malawi  ● Mali  ● Mauritania  ● Mauritius  ● Morocco  ● Mozambique  ● Namibia  ● Niger  ● Nigeria  ● Rwanda  ● Sao Tome and Principe Senegal  ● Seychelles  ● Sierra Leone  ● Somalia  ● South Africa  ● South Sudan  ● Sudan  ● Tanzania  ● Togo  ● Tunisia  ● Uganda  ● Zambia  ● Zimbabwe  *(Multiple choice)* |
| **Question 5**  Geographical location of institution/hospital *  ● Urban  ● Rural  *(Multiple choice)* |
| **Section B**: Neurosurgery exposure of respondents |
| **Question 6**  Have you been exposed to neurosurgery in a formalised clinical rotation? *  ● Yes  ● No  *(Multiple choice)* |
| **Question 7**  If you answered yes to the previous question, what was the length of this rotation? (weeks)  *(Free text)* |
| **Question 8**  What was the nature of this rotation?  ● Dedicated neurosurgery rotation  ● Mixed rotation with other specialties  *(Multiple choice)* |
| **Question 9**  In these rotations, what were you exposed to (either as a participant or observer)?  *Check all that apply.*  ● Exposure to patients managed as inpatients  ● Exposure to patients managed as outpatients  ● Elective surgery  ● Emergency surgery  ● Academic meetings  ● Morbidity and mortality meetings  ● Ward rounds  ● Lectures  ● Bedside tutorials  ● Other: *(free text)*  *(Checkboxes)* |
| **Question 10**  Have you had any other neurosurgery experience outside of a formal clinical rotation? *  *Check all that apply.*  ● Neurosurgery research  ● Neurosurgery webinar  ● Neurosurgery workshop  ● Neurosurgery conference  ● Neurosurgery elective  ● None  ● Other: *(free text)*  *(Checkboxes)* |
| **Question 11**  Does your school have a neurosurgery training program? *  ● Yes  ● No  ● I don’t know  *(Multiple choice)* |
| **Question 12**  Have you had any exposure to neurosurgery outside Africa? *  ● Yes  ● No  *(Multiple choice)* |
| **Question 13**  If yes, what is your take on the quality of neurosurgery in your home country and the country you visited?  *(Free text)* |
| **Section C**: Perception towards a neurosurgical career |
| **Question 14**  The range of operations performed by neurosurgeons is highly diverse  ● Strongly agree  ● Agree  ● Neutral  ● Disagree  ● Strongly disagree  *(Likert scale)* |
| **Question 15**  The future of neurosurgery is bright *  ● Strongly agree  ● Agree  ● Neutral  ● Disagree  ● Strongly disagree  *(Likert scale)* |
| **Question 16**  The outcome of neurosurgical patients is excellent *  ● Strongly agree  ● Agree  ● Neutral  ● Disagree  ● Strongly disagree  *(Likert scale)* |
| **Question 17**  Neurosurgery is very emotionally draining for residents and attendings *  ● Strongly agree  ● Agree  ● Neutral  ● Disagree  ● Strongly disagree  *(Likert scale)* |
| **Question 18**  Neurosurgery residency training is very difficult *  ● Strongly agree  ● Agree  ● Neutral  ● Disagree  ● Strongly disagree  *(Likert scale)* |
| **Question 19**  In the field of neurosurgery, the personalities of attendings and collegiality between faculty is very pleasant and collegial *  ● Strongly agree  ● Agree  ● Neutral  ● Disagree  ● Strongly disagree  *(Likert scale)* |
| **Question 20**  Neurosurgeons are financially secure *  ● Strongly agree  ● Agree  ● Neutral  ● Disagree  ● Strongly disagree  *(Likert scale)* |
| **Question 21**  Neurosurgeons have a good quality of life *  ● Strongly agree  ● Agree  ● Neutral  ● Disagree  ● Strongly disagree  *(Likert scale)* |
| **Question 22**  Becoming a neurosurgeon and having a family is achievable *  ● Strongly agree  ● Agree  ● Neutral  ● Disagree  ● Strongly disagree  *(Likert scale)* |
| **Question 23**  It is more difficult for women to pursue a career in neurosurgery *  ● Strongly agree  ● Agree  ● Neutral  ● Disagree  ● Strongly disagree  *(Likert scale)* |
| **Question 24**  Do you agree that exposure to neurosurgery (or lack thereof) has influenced your perception about a neurosurgical career? *  ● Strongly agree  ● Agree  ● Neutral  ● Disagree  ● Strongly disagree  *(Likert scale)* |
| **Section D**: Interest in a neurosurgical career |
| **Question 25**  Do you want to pursue a career in neurosurgery? *  ● Yes  ● No  ● Maybe  *(Multiple choice)* |
| **Question 26**  You are likely to pursue a career in neurosurgery *  ● Strongly agree  ● Agree  ● Neutral  ● Disagree  ● Strongly disagree  *(Likert scale)* |
| **Question 27**  Would you consider a neurosurgery elective? *  ● Yes  ● No  *(Multiple choice)* |
